# CRISPR-Cas and CRISPR-based screening system for precise gene editing and targeted cancer therapy

**DOI:** 10.1186/s12967-024-05235-2

**Published:** 2024-05-30

**Authors:** Mingming Qin, Chunhao Deng, Liewei Wen, Guoqun Luo, Ya Meng

**Affiliations:** 1 Reproductive Medical Center, Affiliated Foshan Maternity & Child Healthcare Hospital, Southern Medical University (Foshan Women and Children Hospital), Foshan, Guangdong 528000 China; 2 Department of Developmental Biology, School of Basic Medical Sciences, Southern Medical University, Guangzhou, Guangdong 510515 China; 3 Chinese Medicine and Translational Medicine R&D center, Zhuhai UM Science & Technology Research Institute, Zhuhai, Guangdong 519031 China; 4 Guangdong Provincial Key Laboratory of Tumor Interventional Diagnosis and Treatment, Zhuhai People’s Hospital, Zhuhai Clinical Medical College of Jinan University, Zhuhai, Guangdong 519000 China

**Keywords:** CRISPR-Cas system, CRISPR screening, Cancer therapy, CAR-T, Precision medicine

## Abstract

Target cancer therapy has been developed for clinical cancer treatment based on the discovery of CRISPR (clustered regularly interspaced short palindromic repeat) -Cas system. This forefront and cutting-edge scientific technique improves the cancer research into molecular level and is currently widely utilized in genetic investigation and clinical precision cancer therapy. In this review, we summarized the genetic modification by CRISPR/Cas and CRISPR screening system, discussed key components for successful CRISPR screening, including Cas enzymes, guide RNA (gRNA) libraries, target cells or organs. Furthermore, we focused on the application for CAR-T cell therapy, drug target, drug screening, or drug selection in both ex vivo and in vivo with CRISPR screening system. In addition, we elucidated the advantages and potential obstacles of CRISPR system in precision clinical medicine and described the prospects for future genetic therapy.

In summary, we provide a comprehensive and practical perspective on the development of CRISPR/Cas and CRISPR screening system for the treatment of cancer defects, aiming to further improve the precision and accuracy for clinical treatment and individualized gene therapy.

## Introduction

**Fig. 1:**
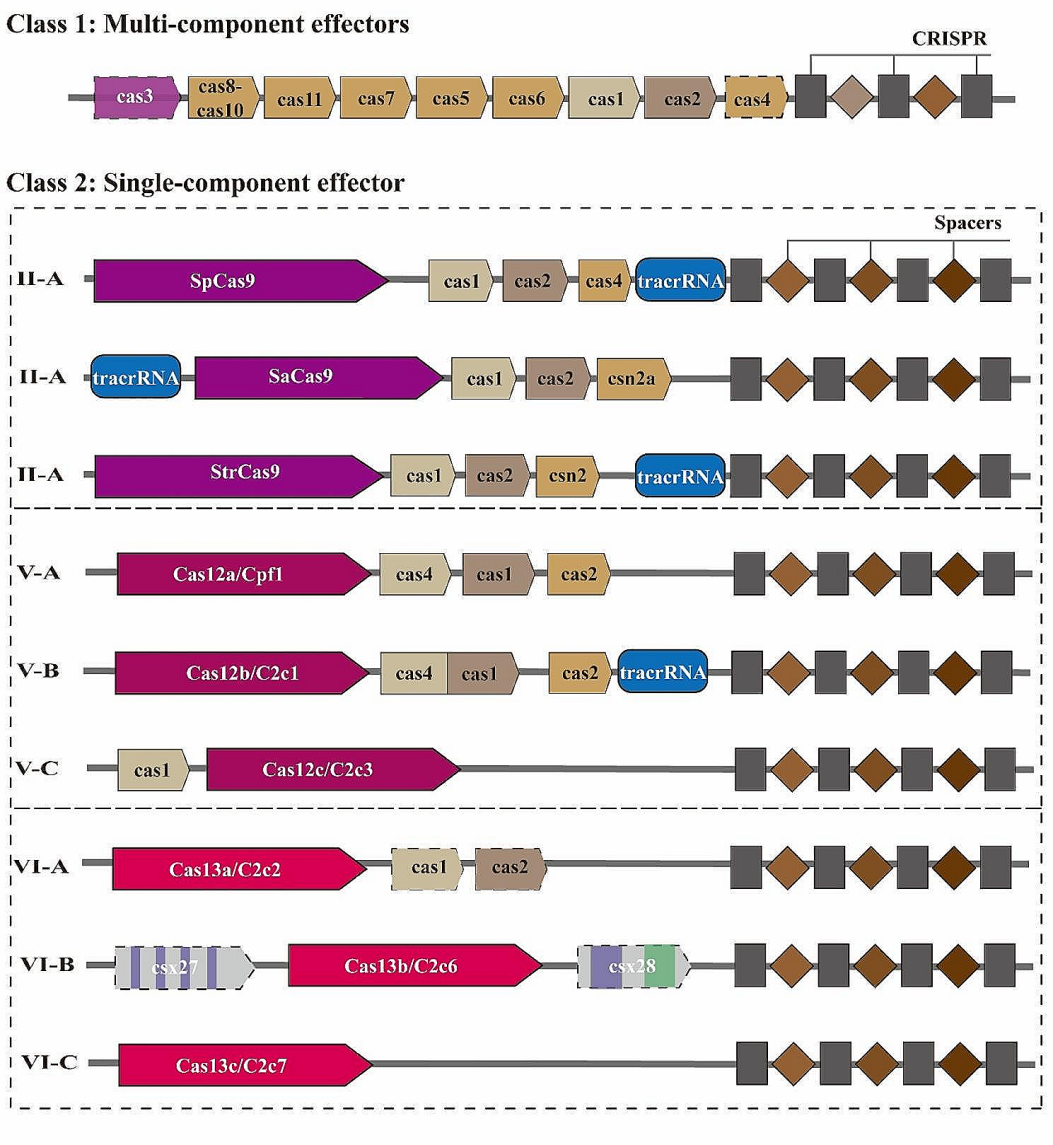
Schematic representative of CRISPR/Cas loci in Class 1 and Class 2 system. Class 1 system show multi-component effectors, while the Class 2 system have one effector. Three subgroups of Class 2 CRISPR systems are presented. Representative Type II-A CRISPR protein contains: *Streptococcus pyogenes* Cas9 (SpCas9), *Staphylococcus aureus* Cas9 (SaCas9) and *Streptococcus thermophilus* Cas9 (StrCas9), all of which have the tracrRNA sequences. Type V CRISPR, which comprises Cas12a, Cas12b and Cas12c, exhibits distinct genome structures. Cas12b has the tracrRNA structure, while Cas12c only has one assistant protein cas1 for genome editing. Cas14 subgroup is not depicted in this figure. Type VI CRISPR systems show few assistant proteins to identify RNA virus, however,  type VI-B has csx27 and csx28 proteins to regulate nuclease activity. Illustrated according to Ref [14, 16, 20, 21].

Cancer therapy has been developed from the very initial surgical removal in the ancient to currently precision minimally invasive surgery; from the chemotherapy, radiotherapy to the targeted therapy and precision individualized immunotherapy, under the progress of precise and granular molecular characterization at present [1]. The newly discovered genome editing tool CRISPR (clustered regularly interspaced short palindromic repeat) /Cas system provides a powerful method for the investigation of cancer therapy [2–4]. It was described initially in bacteria as a primitive immune system to fight against viral infections and was universally recognized as a genomic modification system in the past decade [5, 6]. In Prokaryotes, the short DNA repeats CRISPR exist between regular spacing units, and are recognized as intervening sequences derived from preexisting fragment of bacteriophages and conjugative plasmids, contributing to bacteria immune system [7]. The genetic sequences of the viral invaders or plasmid challengers are captured and aligned as spacer segments in the CRISPR region in bacteria or archaea [8, 9], comprising the CRISPR-mediated adaptive immunity system [10]. Two classes of CRISPR-Cas systems have been described in prokaryotes based on their effector modules [11–14], characterized into 6 types, and 33 subtypes described in 2020 [15]. The Class 2 CRISPR-Cas system composed only 10% percentage but has expanded biotechnology toolbox for genome editing with 190,000 shares worldwide from 640 labs [16, 17]. It consists of three types of effectors: type II, type V and type VI, with several widely recognized genetic editing enzymes, being Cas9 in type II, Cas12a (Cpf1), Cas12b (C2c1), Cas12c (C2c3), Cas14 subgroup in type V [18], Cas13a (C2c2), Cas13b (C2c6) and Cas13c (C2c7) in type VI [14, 19]. Schematic representation of two classes of CRISPR/Cas systems were depicted in Fig. 1.

CRISPR/Cas system has been utilized for cellular genetic modification [22, 23] and the generation of animal models for cancer research [24, 25]. Furthermore, the CRISPR/Cas-based genetic screening system was developed for cellular investigation [26–28], as well as in tumor studies [25, 29]. In addition, high throughput gRNA libraries have been established to enable efficient genetic screening, specially facilitating personalized treatment strategies for cancer patients individually [30]. In this review, we provide a comprehensive overview of the CRISPR/Cas system and essential elements for successful CRISPR screening system, including gRNA libraries, gRNA validation, and clinical application for cancer research. Furthermore, we explored the application of the CRISPR screening system in cancer therapy from both ex vivo and in vivo investigation, aiming to elucidate the inherent advantages and potential obstacles for clinical precision medicine.

## The application of Class 2 CRISPR-Cas effectors and genome modification in cancer therapy

### Type II effector Cas9 in cancer research

Both *Streptococcus pyogenes* Cas9 (SpCas9) and *Staphylococcus aureus* Cas9 (SaCas9), classified as the type II-A effectors, showed comparable genome editing efficiency for in vitro and in vivo study [21, 31–33]. These effectors enable rapid modification of cellular or animal models for transcriptional modulation via CRISPR knockout/knockin or high throughput genomic screening [23, 34]. The compact size of SaCas9 renders it an optimal enzyme for in vivo AAV application. However, SpCas9, one of the pioneering Cas9 proteins, has been extensively investigated and utilized in CRISPR gene editing. Three variants of SpCas9 have been developed, the wild-type Cas9, nickase Cas9 (nCas9), and dead Cas9 (dCas9).

Cas9 mediated DNA cleavage with the two distinct active sites RuvC and HNH, under the assistance of CRISPR RNA (crRNA) and trans-activating crRNA (tracrRNA) ribonucleoprotein complex [8]. The dual-tracrRNA: crRNA chimera single guide RNA (sgRNA) was created and directed Cas9 nuclease to the potential target loci for site-specific DNA cleavage, initiating the genome editing system* in vitro* [35]. The binding of Cas9 to the adjacent sequence of three nucleotides, known as protospacer adjacent motif (PAM), triggers DNA cleavage by inducing double-strand breaks with its scissor-like activity [36]. The recently used Cas9-gRNA ribonucleoprotein (RNP) complexes remarkably increase fidelity and efficacy for double-strand DNA breaks with minimized cell mortality [37]. It also combined with repair donor to achieve site-specific correction of cystic fibrosis transmembrane conductance regulator (CFTR) gene mutations in epithelial organoids [38]. Cre-dependent Cas9 knockin mouse was generated, and *KRAS, p53*, and *LKB1* depletion resulted in carcinoma formation in these transgenic mice, providing a robust cancer model for research [24].

One mutation in D10A of Cas9 protein makes a nCas9, which improves genome editing specificity [39]. The combination of sgRNA pairs with nCas9 significantly enhances cutting specificity by 50-1000 folds in cell lines and mouse zygotes [40]. CRISPR-Cas base editing using nCas9 enables precise incorporation of point mutations in genomic DNA without inducing double-strand breaks, demonstrating its potential in treating genetic diseases caused by base-pair alterations through adenine base editors (ABEs) or cytosine base editors (CBEs) [41]. In addition, DNA base editors combining with the leading platform adeno-associated virus (AAV) vector for viral delivery expanded the CRISPR-base-edit toolkit for Prime-editing (PE) [42]. Meanwhile, the recently developed genome editing technique known as NICER utilizes Cas9^D10A^ nickase to correct heterozygous mutations. It generates multiple DNA nicks and triggers gene correction via interhomolog homologous recombination (IH-HR) which rarely induces genomic alterations, making it a precise strategy to restore genetic diseases or single nucleotide mutations [43]. Except the precise single nucleotide restoration, cancer translocations were generated by double strand breaks and paired nicks with either Cas9 or nCas9, creating endogenous chromosomal translocations cell model for investigating tumor driving genes [44].

**Fig. 2:**
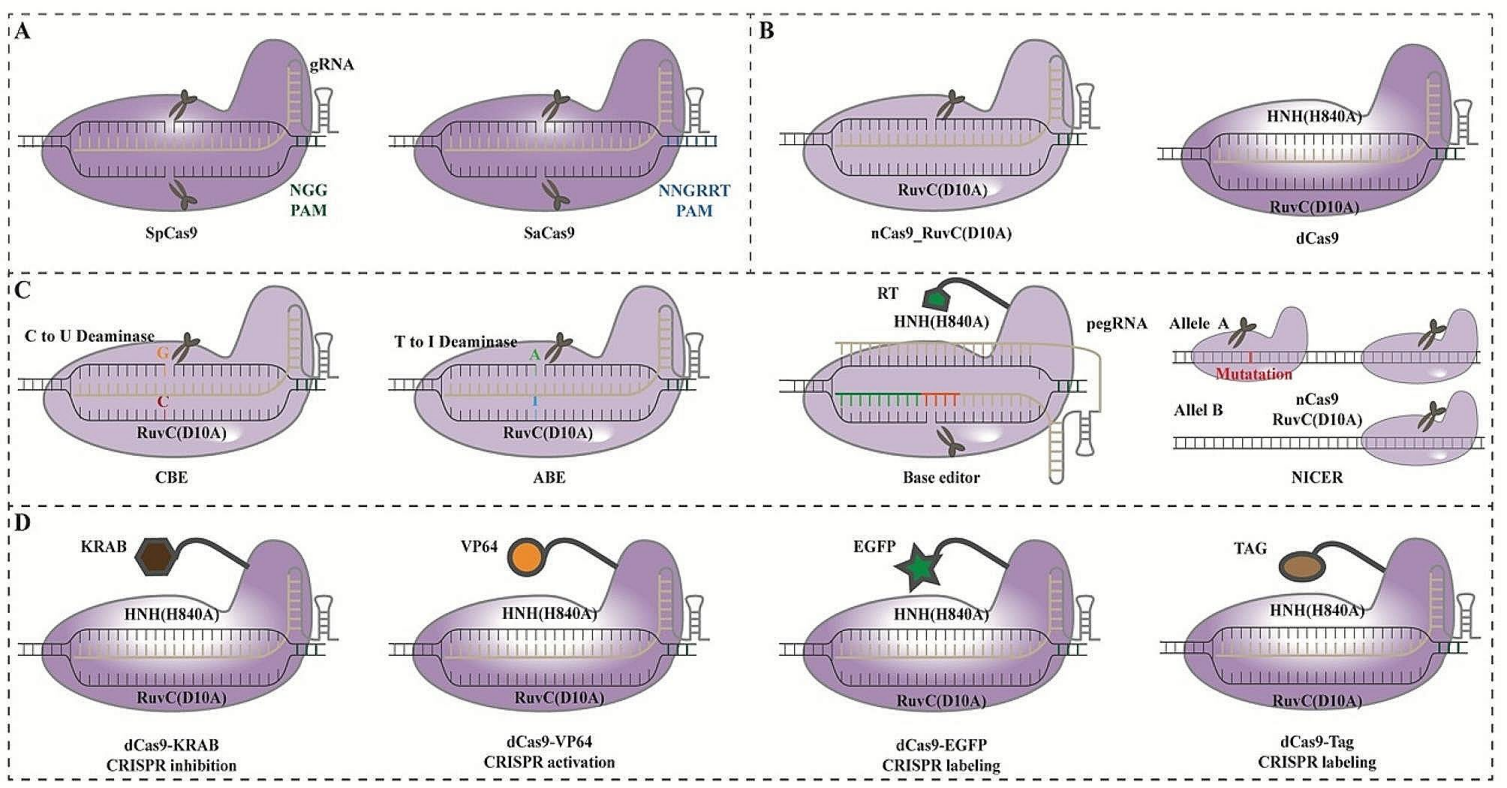
Summary of Cas9 proteins and modified nCas9 and dCas9 genome editing tools. **(A)** PAM for SpCas9 is NGG, while PAM for SaCas9 is NNGRRT with the ability to cut DNA double helix. **(B)** Mutation of D10A leads to the formation of nCas9 while both mutations generate dCas9 protein. **(C)** nCas9 can be applied for base editing such as CBE and ABE, also for Base editor and developed as NICER to repair heterogenous mutation. **(D)** dCas9 was modified to generate CRISPRi, CRISPRa and CRISPR labeling tools. dCas9: dead Cas9. nCas9: nickase Cas9. CBE: Cytosine Base Editor, ABE: Adenine Base Editor. RT: reverse transcriptase. pegRNA: prime editing guide RNA.

Catalytically inactive Cas9, a ‘dead’ protein (dCas9) with both mutations in D10A and H840A of RuvC and HNH domains, showed its popularity in gene regulation with inhibition, activation, and cell imaging and labeling [45]. Genome-scale screenings utilizing CRISPR inhibition (CRISPRi) and CRISPR activation (CRISPRa) have been employed to identify both known and novel genes involved in controlling cell growth and sensitivity to toxins [46]. Precise inducible gene knockdown or overexpression can be supported using dCas9-KRAB (Krüppel-associated box) or Cas9 combined with Tetracycline Inducible Expression promoter (TetO) [47]. Firstly, the fusion of dCas9 with transcriptional repressor produces the CRISPRi genetic tool [48]. The dCas9-BFP-KRAB repressor domain enables the suppression of gene expression [49]. Second, fusing dCas9 with RNA polymerase (RNAP) omega subunit upregulates gene expression [50], and dCas9-VP64 was used for transcriptional activation [51]. In addition, dCas9 protein serves as a valuable tool for labeling of endogenous genomic loci in living cells. By employing an optimized sgRNA fused with EGFP-tagged dCas9, repetitive elements in telomeres and various other regions can be robustly labeled [52]. A double-color CRISPR labeling method was established by incorporating MS2 or PP7 RNA aptamers into the sgRNA, fused with the catalytically inactive Cas9 (dCas9) for direct visualization [53]. Finally, dCas9 can be employed for in vivo imaging of chromosomal dynamics and genome organization dimensions [47], allowing systematic fluorescent labeling of up to 10 proteins [48]. Summary of the type II Cas9 enzymes was depicted in Fig. 2.

### Type V and type VI effectors in cancer research

Mainly three subtypes of type V effectors were investigated for gene editing, named as type V-A, V-B and V-C. The type V-A effector Cpf1 (CRISPR from *Prevotella* and *Francisella 1*), exhibits enhanced genome editing specificity attributed to a T-rich PAM (-5′TTTV) [54], resulting in a staggered DNA double stranded break [55]. Two candidate Cpf1 (Cas12a) enzymes, AsCpf1 from *Acidominococcus sp. BV3L6* and LbCpf1 from *Lachnospiraceae bacterium ND2006*, show a robust genome editing ability in human cells compared to that of Cas9 [56]. Furthermore, successful generation of gene knockout transgenic mice was achieved using both AsCpf1 (40.7%) and LbCpf1 (28.6%), providing a wonderful animal model for research [57, 58]. Multiplex genome editing was conducted using Cpf1 from *Aspergillus aculeatus* strain TBRC277 [59] and AsCpf1 was engineered with adeno-associated viral vectors (AAVs) for multiplex genome editing of mouse brain in vivo [60]. One-step generation of homology-directed repair (HDR) and checkpoint knockout CAR-T (KIKO CAR-T) was achieved with the adeno-associated virus and CRISPR/Cpf1 system, establishing an efficient AAV-Cpf1 double knockin system and opening new possibilities for cancer research [61]. The type V-B CRISPR effector Cas12b (C2c1) discovered in *Bacillus hisashii* (BhCas12b) showed a nickase effect at 37 °C for human gene editing, while BhCas12b v4, containing K846R/S893R/E837G mutants, demonstrated strong genome editing ability in human cells comparable to SpCas9 [62]. While the type V-C CRISPR effector Cas12c (C2c3) is a site-specific ribonuclease generating mature crRNAs for DNA targeting, crRNAs direct DNA binding by Cas12c without DNA cutting, providing a DNase-free pathway for transient antiviral immunity [63].

While both type II and type V are effective for DNA targeting in the genome level, the type VI effector Cas13 exhibits efficacy in treating genetic diseases and rescuing diseased sequences at the RNA level. They provide valuable genetic tools for diagnosis and degradation of viruses such as HIV and HPV [64, 65]. Several Cas13 proteins were characterized, such as Cas13a, Cas13b, Cas13bt and Cas13d, showed the efficiency to cleave single stranded RNAs [66–68]. Of which Cas13a based SHERLOCK (Specific High-Sensitivity Enzymatic Reporter UnLOCKing) system can detect Zika or Dengue Virus as well as somatic mutations in cell free DNA (cfDNA) samples such as serine/threonine kinase (BRAF) V600E cancer mutation [69]. Shortened detection time and high sensitivity were applied for virus detection via SHERLPCKv2 system [70, 71].

SHERLOCK enables to identify EGFR-T790M mutation in patient DNA with high efficiency by detecting 0.6% mutant ratio samples [72], this system was also used for DNA and RNA detection with single-base specificity and attomolar sensitivity in cancer patients samples [73]. Cas13b was used to fight RNA viruses such as porcine reproductive and respiratory syndrome virus (PRRSV) [74], chikungunya (CHIKV) and dengue in mosquito cells [75] as well as SARS-CoV-2 resistance [76, 77]. Since Cas13b targets RNA without interfering genome sequence of the targeted gene, it provides a potential safer alternative to Cas9 enzymes. Catalytically inactive Cas13b (dCas13b) was engineered to direct adenosine-to-inosine deaminase for precise base editing, enabling the Programmable A to I Replacement (REPAIR) RNA editing platform. This platform can be utilized in transcriptome engineering of advanced leukemias, as well as head, liver, and breast cancers, thereby demonstrating a feasible strategy for investigating gene function in cancer at the RNA level [78, 79]. The RNA-targeting CRISPR-Cas13 system showed promising roles in cancer diagnosis, therapy, and research; with the ability for early detection of cancer markers in liquid biopsy samples, degradation and manipulation of cancer-related mutant transcripts, as well as identification of novel therapeutic drug targets described in the recent review [80].

Altogether, the class 2 effectors expanded the current CRISPR/Cas toolkit. Cas9 possesses recognition ability of specific target sequences, and has the genomic editing ability for precision cancer treatment and mutation detection [2]. Meanwhile, the recently discovered Cas12 and Cas13 expand RNA editing tool, providing novel genetic methods for cancer diagnosis and molecular examination of cancer research [3].

## The application of CRISPR screening system in cancer

**Fig. 3:**
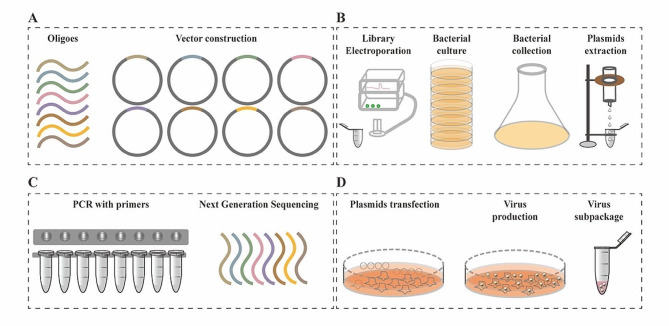
Schematic representation of gRNA library construction and virus production. **(A)** Oligoes synthesis and vector construction for gRNA library. **(B)** Amplification of gRNA library by bacterial culture, collection, and plasmid extraction. **(C)** PCR examination and sequence confirmation for library coverage. **(D)** Plasmids transfection and virus production with a certain gRNA library.

The development of CRISPR/Cas system and high-throughput sequencing makes genetic screening easily accessible in basic biology, drug discovery, and personalized medicine for cancer therapy [3]. Cas9 nuclease is a preferred choice for genetic screening, and has been used for genomic modification in multiple researches [26, 28, 81, 82]. One-step generation of multiplex genome mutations via CRISPR/Cas9 system was successfully achieved in mice, facilitating in vivo functional analysis of redundant genes [83]. CRISPR screening system was developed based on CRISPR/Cas combined with thousands of gRNAs integrated into viral vectors [81, 84]. These libraries harbor gRNAs targeting various genes, and have received up to 1000 annual requests globally, enabling unbiased, phenotypic forward genetic screening [17]. The first whole genomic gRNA libraries for both mouse and human were generated with mouse lentiviral gRNA library containing 87,897 gRNAs for 19,150 coding genes, naming as (GeCKOv1), and was established to screen out unknown genes for *Clostridium septicum* alpha-toxin or 6-thioguanine (6TG) drug resistance [81]. However, low viral titer of the lentiviral delivery systems in GeCKOv1 limited the usage for biological screening, and genome-scale CRISPR knockout v2 (GeCKOv2), contained 123,411 unique sgRNAs targeting 19,050 annotated protein-coding genes and 1000 control sgRNAs (sg-NTCs), resulting in a 10-fold increase for viral generation [84]. Optimized mouse gRNA libraries targeting 20,611 genes with 130,209 gRNAs were also established with 100-fold increase of functional viral titer [84]. Innovative strategies of CRISPR-Cas9 system have been developed for large-scale genome knockout and transcriptional activation [85], as well as combinatorial genetic screening [27]. Processes for gRNA library generation and amplification were illustrated as depicted in the following Fig. 3.

### gRNA libraries for cancer research

Various of genome-scale gRNA libraries were established for CRISPR screening, and some gRNA libraries for specific selected genes were also established with small capacity. Established gRNA libraries of genome wide and specific selected targets for cancer research were summarized in the Table 1.

Human lentiviral GeCKOv1 library (lentiCRISPRv1) was established for high throughput gene targeting of 18,080 genes, with 64,751 unique gRNAs total, and was used for cell viability-related gene screening in cancer. It was also examined for resistance to a therapeutic RAF inhibitor, vemurafenib, in a A375 melanoma model, leading to the discovery of novel genes sensitive to drug treatment [28]. GeCKOv2 library was also used to identify responsible genes related to EGF-induced apoptosis [86]. Genome-wide sgRNA library (mGeCKOa) transfection in non-metastatic mouse non-small cell lung cancer with 67,405 sgRNAs targeting 20,611 protein-coding genes. Cells were treated and transplanted into immunocompromised Nu/Nu mice, and tumor growth and migration were evaluated in vivo [25]. The pooled lentiviral sgRNA library with 73,151 gRNAs targeting 7114 gene and 100 non-targeting controls were used to screen the resistant genes for nucleotide analog 6TG treatment in human leukemic cell lines, screening resistance genes toward chemotherapeutic etoposide [26]. Patient-derived glioblastoma cell line (GBM), retinal epithelial cells (RPE1), colorectal carcinoma (HCT116 and DLD1), cervical carcinoma (Hela) and melanoma (A375) cells were subjected into genetic screening with the “90k library” containing 17,232 targeting genes and 91,320 gRNA sequences. Subsequentially, the supplemental library naming 176,500 TKO (Toronto KnockOut) library targeting 17,661 protein-coding genes were used to identify fitness genes in cancer cell lines [87]. Lentiviral vectors with genome-scale sgRNA library consisting of 70,290 guides (3 sgRNAs for each transcription start site (TSS)) were used for synthetic activation mediator (SAM)-based screening to target 200 bp upstream of the TSS and confer resistance to a BRAF inhibitor in melanoma cell line A375 and patient derived samples [51].

Although genome scale gRNA libraries are widely used in cancer research, its complexity and transcript isoform variance as well as difficulty in viral vectors cloning limited its usage. Other specific gRNA libraries for certain signal pathways or gene functions were established according to screening purpose for modulating endogenous genes. Total 5920 candidate enhancers were perturbed by the dCas9-KRAB enzyme, establishing the multiplex, expression quantitative trait locus (eQTL) framework, and total 664 cis enhancer-gene pairs were identified and enriched based on 254,974 single-cell transcriptomes in K562 derived from a chronic myologenous leukemia patient [49]. Undescribed immunotherapy targets for transplantable melanoma tumors in mice were explored with the 9992 sgRNAs targeting 2368 genes selected from transduced cells, establishing the in vivo genetic screen tumor models [88]. Recurrently mutated genes derived from pan-cancer The Cancer Genome Atlas datasets were recognized as well-known tumor suppressors genes (TSGs) or oncogenes. Total 49 orthologs of human TSGs were found in mouse genome, and the mouse TSG library containing 280 sgRNAs targeting 56 different genes (7 housekeeping genes) were used for tumor metastasis analysis [89].

**Table 1:** gRNA libraries for cancer research

Species	Vector type	Library capacity	gRNAs number	Target cells	Modification type	Reference
Mouse	Lentiviral	19,150	87,897	male ESCs	CRISPR KO	[81]
Human	Lentiviral	18,080	64,751	A375 HUES62	CRISPR KO	[28]
Human	Lentiviral	7,114	73,151	KBM7 HL60	CRISPR KO	[26]
Mouse	AAV	49	278	Liver	CRISPR KO	[89]
Human	Lentiviral	1,119	5,920	K562	CRISPRi	[49]
Human	Lentiviral	23,430	70,290	A375	CRISPRa	[51]
Human	Lentiviral	17,232	91,320	DLD1 HCT116 RPE1 HeLa GBM A375	CRISPR KO	[87]
		17,661	176,500			
Human	Lentiviral	19,050	123,411	A549 A431 HEK293FT	CRISPR KO	[86]
Mouse	Lentiviral	20,611	67,405	NSCLC	CRISPR KO	[25]
Mouse	Lentiviral	2,368	9,992	B16	CRISPRi	[88]
Mouse	Lentiviral	21,786	125,793	BMDCs	CRISPRko	[90]

### The improvements of specificity and validation methods for gRNA Library

The procedure to perform pooled genome-editing experiments was clearly described, and successful CRISPR/Cas9 screening needs the specific and efficient gRNA sequence with proper quality and low off-target effect [91]. Off-target predictions calculated by algorithms indicating false positives and quantified error rates were developed by Bowtie and BWA sequencing methods, or considered by MIT-Broad score and the CFD score as summarized in previous reviews [92]. Computational tools for sgRNA designing with low off-target and high on-target efficacy and specificity have been developed and summarized in 2018 [93]. Several methods have been built for eliminating off-target results such as the utilization of high-efficiency delivery RNP tool, modification of the gRNA sequence, and improvement the specificity of Cas9 Enzymes [94]. The computational tool CRISPOR established high-quality gRNA libraries by selection according to off-target and on-target predictions, it also helps with vector cloning, gRNA validation and expression with primer designing and restriction enzymes depiction [95]. Optimized on-target efficiency prediction model was generated to illustrate the cleavage ability of gRNA sequence (http://crispor.org) [96]. Meanwhile, CRISPResso provides a robust and user-friendly computational pipeline to evaluate effects of coding and noncoding sequences and select off-target sites [97]. For precise gene selection analysis, the Model-based Analysis of Genome-wide CRISPR/Cas9 Knockout (MAGeCK) is the optimized method for both positive and negative selection, which offers high sensitivity and low FDR regardless of sequencing depth or sgRNA numbers for a single gene [98]. Besides that, intergration deficient lentiviral (IDLV) capture [99], and high-throughput genome-wide translocation sequencing (HTGTS) [100] are other methods for off-target detection.

Analysis of gRNAs abundance in pooled libraries plays an important role in targeting efficiency and screening accuracy and specificity. PCR products of gRNA library vectors can be sequenced on Hiseq 2500 and aligned to sgRNAs by Bowtie, an ultrafast, efficient program for aligning short DNA sequence to large genomes [101]. Rigorous analytical methods mitigate the false discovery rates generated by CRISPR screens via a Bayesian classifier of gene essentiality [102]. Sequence quality control can also be carried out under the guide of GPP Pooled Screen Analysis (https://portals.broadinstitute.org/gpp/broad/), and statistical enrichment and gene depletion were calculated by hit calling algorithm STARS (http://www.broadinstitute.org/rnai/public/software/index) based on normalized fold changes [103]. High-content downstream gRNA library sequence validation in tumor immunology were summarized in the recent review [29]. Generally speaking, breaks labeling, enrichment on streptavidin and next-generation sequencing (BLESS) [104], genome-wide unbiased identification of DSBs enabled by sequencing (GUIDE-seq) [105, 106] and discovery of in situ Cas off-targets and verification by sequencing (DISCOVER-seq) [107–109] were used as cell based methods with direct sequencing. More sensitive biochemical methods such as digested genome sequencing (Digenome-seq) [110–112], selective enrichment and identification of adapter-tagged DNA ends by sequencing (SITE-Seq) [113], circularization for in vitro reporting of cleavage effects by sequencing (CIRCLE-seq) [114, 115] and circularization for high-throughput analysis of nuclease genome-wide effects by sequencing (CHANGE-seq) [116] were developed for accurate sequence confirmation.

## CRISPR screening application in cancer therapy

**Fig. 4:**
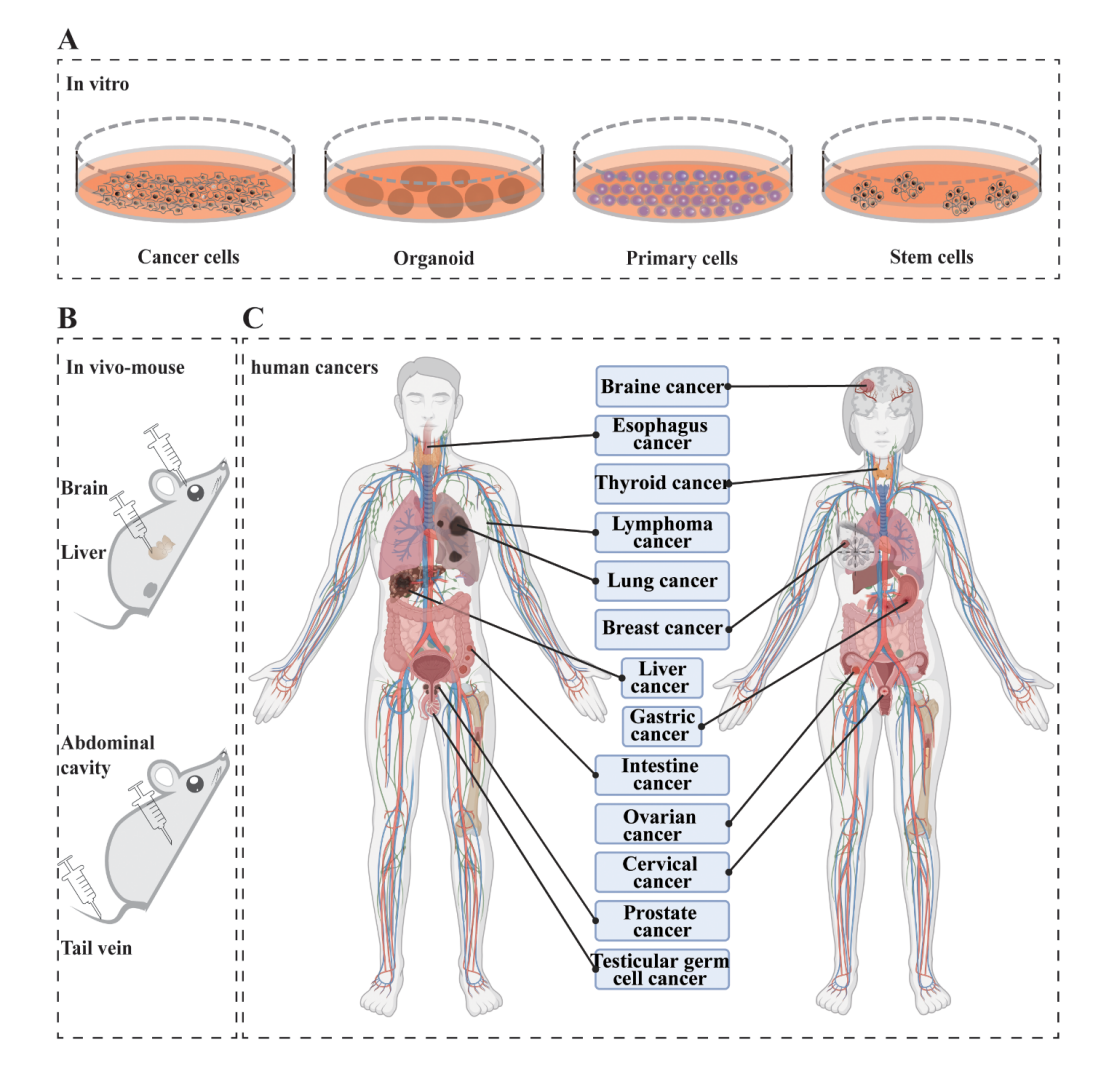
CRISPR screening and its applications in ex vivo and in vivo for cancer therapy. **(A)** CRISPR screening application in cultured cells. **(B)** CRISPR screening in vivo application in mouse with direct injection to organs and indirect injection in abdominal and tail vein. **(C)** Schematic representation of CRISPR screening applications for human cancers; Created with BioRender.com

The application of the CRISPR/Cas system for cancer therapy has been investigated using viral vectors including lentivirus, adenovirus, and AAV vectors, as well as non-viral vectors such as polymer nanoparticles, golden nanoparticles, or lipid nanoparticles in both ex vivo and in vivo circumstances as described in recent reviews [117, 118]. Various cancer cell lines [2, 4, 87, 119, 120], T-cells via chimeric antigen receptor (CAR) integration or CAR-T system [90, 121, 122], and organoids derived from patient samples [123] have been explored for cancer therapy research. However, because of manipulation limitations in highly differentiated cells, in vivo clinical precision therapy involving modified cells with AAV vector delivery for the CRISPR modification system is widely used for a broad range of human diseases [118]. In this part, we mainly focus on the application of CRISPR screening system for cancer therapy, including ex vivo and in vivo approaches. Schematic representation of CRISPR screening applications for cancer research is summarized in Fig. 4.

### CRISPR screening in vitro for cancer therapy

CRISPR screening has several potential applications in cancer therapy, including modified T cells and Chimeric antigen receptor CAR-T cancer treatment, novel target identification, drug resistance, drug selection exploration and so on [4, 29]. The CRISPR screening system has been employed to investigate various cancer cell types originating from diverse organs including lymphatic system, esophagus, stomach, intestines, lungs, nervous system, skin, liver, blood cells as well as reproductive organs. CRISPR screening applications in Cancer therapy were summarized in Table 2.

**Table 2:** CRISPR screening ex vivo for cancer research

Cas enzyme	gRNAs library	Target cells	Screening	Application	Reference
Cas9	Addgene #73,179	CAR-T cells Glioblastoma stem cells	CRISPRko	Gene dependencies	[124]
Cas9	Addgene #52,961	CD19 + NALM6 B-ALL cells	CRISPRko	Drug sensitivity	[125]
Cas9	Addgene #52,961	Esophageal squamous cell carcinoma (ESCC)	CRISPRko	cancer metastasis driver genes	[126]
Cas9	Addgene # 51,047	Pancreatic ductal adenocarcinoma cells	CRISPRko	Drug targets	[127]
Cas9	Addgene #52,961	Hepatocellular carcinoma cells	CRISPRko	Metabolic vulnerabilities	[128]
Cas9	Addgene #108,098, #124,772	Epithelial ovarian cancer(EOC) cell lines	CRISPRko	Drug resistance genes	[129]
Cas9	Addgene #52,962, #73,178	GNAQ-mutant UM cells	CRISPRko	Gene dependencies	[130]
Cas9	TSG dual gRNA library	MCF10A cells MCF10A PTEN-/- cells MCF10A-PIK3CA MCF10A-MYC	CRISPRko	Tomor suppressor genes	[131]
Cas9	(GeCKO v2)	Small-cell lung cancer SCLC cell lines	CRISPRko	Chemotherapy resistance genes	[132]
Cas9	Addgene #67,989	Human AML cell ines	CRISPRko	Therapeutic targets	[133]
Cas9	Addgene#164,896	Human liver cancer cell lines	CRISPRa	Driver genes for carcinoma growth	[134]
dCas9-KRAB	-	Prostate cancer	CRISPRi	Gene dependencies	[135]
Cas9	(GeCKO v2)	Nasopharyngeal carcinoma cell lines	CRISPRko	radiosensitive and radioresistant genes	[136]
Cas9	Self-constructed gRNA	Neuroblastoma	CRISPRko	Novel targets	[137]
Cas9	Addgene #1,000,000,048	Human ovarian carcinoma	CRISPRko	Drug resistance	[138]
Cas9	Addgene #73,179	Hela/SiHa cells	CRISPRko	Drug resistance	[139]
Cas9	Addgene #52,962	C4, LNCaP, PC3, and DU145 cells	CRISPRko	Drug resistance	[140]
Cas9	sgRNA library for Kinases genes	MDA-MB-231, SKBR3, and MCF7	CRISPRko	Drug resistance	[141]
Cas9	sgRNAs library targeting 565 E3 ubiquitin ligase genes	PCa DU145 cells	CRISPRko	Drug resistance	[142]
Cas9	Addgene #73,179	CRC cell line SW480 CRC organoids	CRISPRko	Drug resistance	[143]
dCas9-VP64	The Human CRISPRa library (SAMv2)	HANK-1 and NK-92 cell lines	CRISPRa	Radiotherapy resistance	[144]
dCas9-VP64	Human CRISPR activation plasmid library (SAMv2)	Testicular germ cell tumors	CRISPRa	Drug resistance	[145]
Cas9	Human CRISPR Library Yusa v.1.1	Colorectal cancer organoid HCM-SANG- 0266-C20	CRISPRko	Drug sensitivity testing	[146]
Cas9	Addgene #47,108	Human fetal hepatocyte organoid lines	CRISPRko	Drug screening	[147]
Cas9	Addgene #83,480	Human PDAC organoid	CRISPRko	Drug gene interaction	[148]
Cas9	Addgene #51,043	HL60 pseudo-diploid human leukemic cell line KBM7 CML cell line,	CRISPRko	Drug resistance	[26]
dCas9-KRAB	Addgene #106,280	Chronic myelogenous leukemia cell line K562	CRISPRi	Gene regulation	[49]
Cas9	Addgene #1,000,000,048 Addgene #1,000,000,049	EGFR-overexpressing cancer A431/A549 cell lines	CRISPRko	Drug resistance	[86]
Cas9	Self-designed gRNA library	Murine acute myeloid leukemia cells	CRISPRko	Drug target	[119]

#### Modified T cell and CAR-T therapy for cancer therapy

Immune system is the most important defender to fight off cancer. Immunotherapy strategy is to make better immune cells such as tumor-infiltrating lymphocytes (TIL) or CAR-T cells to attack cancer via T-cell transfer. TIL therapy uses patient’s own lymphocytes to kill tumor, whereas CAR-T means modified T cells with specific proteins from surface of cancer cells, thus having the ability to attack tumors. In addition to Cas9 utilization, conjugated Cas12 (cCas12a) can be used for CAR-T cell generation. Using an AAV vector, Cas12a-crRNA complex showed robust efficiency to generate site-specific and precisely targeted CAR-T cells [149].

Recent review showed the importance of gamma retroviral or lentiviral vectors for CAR-T cell generation to target B-cell lymphomas and leukemias, although with complex manufacturing procedure, providing a promising “off-the-shelf” products for cancer treatment [150]. Whole-genome CRISPR/Cas9 screening was performed in CAR-T cells and co-cultured with Glioblastoma (GBM) stem cells (GSCs) to explore the PD-1 dependence genes such as *TLE4* and *IKZF2* for cancer treatment. Meanwhile, transduced GSCs were subjected to CAR-T challenge in order to identify enriched and depleted genes for cancer cell apoptosis [124]. Until 2021, total 3 FDA approved CAR-T therapies have been described as *tisagenlecleucel, axicabtagene ciloleurel*, and *brexucabtagene autoleucel* based one CD19-mediated CAR-T cells [151]. Although CAR-T is efficient in blood cancers, its efficiency loss impedes the treatment efficiency. To overcome refractory of B-cell malignancies, genome-scale CRISPR-Cas9 loss-of-function screens were performed, and revealed the crucial role of FADD and TNFRSF10B (TRAIL-R2) in mediating CAR-T cell cytotoxicity [125]. Except for precision CAR-T treatment, multiplexed CRISPR-Cas9 editing applications have been used to generate universal CAR-T products, with the aim of enhancing antitumor efficacy and improving safety of cell-based therapies [152].

#### Novel targets identification using CRISPR/Cas9 screening in cancer research

The invasion and metastasis of cancers make it more difficult to treat, and new targets should be identified for complete cure. Using genome-wide CRISPR/Cas9 screening, key drivers for invasion and metastasis of esophageal squamous cell carcinoma (ESCC) were identified by gain- and loss-of-function experiments, demonstrating that high expression of Mesoderm Specific Transcript (MEST), interacting with purine rich element binding Protein A, is associated with poor patient survival via activating SRCIN1/RASAL1-ERK-snail signaling [126]. Synergistical effect of genetic deletion and pharmacologic inhibition to increase cytotoxicity of MEK signaling inhibitors in pancreatic ductal adenocarcinoma cells was also investigated by CRISPR knockout screening [127]. Genome wide CRISPR/Cas9 knockout screening identified Zinc finger protein (ZNF) family member ZNF319 as a potent suppressor responsible for metastasis of breast cancer in an orthotopic murine model [153]. In hepatocellular carcinoma (HCC), CRISPR/Cas9 knockout library screening revealed the crucial role of pyruvate metabolism in HCC treatment, particularly when combined with a glutamine-deficient diet, showing the targetable metabolic vulnerabilities of pyruvate dehydrogenase α(PDHA), pyruvate dehydrogenase β(PDHB), and pyruvate carboxylase (PC) [128]. CRISPR-Cas9 knockout mutagenesis to exons encoding functional protein domains was performed to screen drug targets and dependencies, providing a comprehensive identification of protein domains for cancer cell sustainment [120]. In epithelial ovarian cancer (EOC), CRISPR-Cas9 screening combined with olaparib treatment successfully identified five genes, ATM, NBN, MUS81, RAD51B, and BRCA2, as predictive markers for olaparib response. Additionally, CDK12 emerged as a promising therapeutic target for EOC without compromising the efficacy of Olaparib response [129]. The whole-genome CRISPR screening in Guanine nucleotide-binding protein G(q) subunit alpha (GNAQ) mutant uveal melanoma (UM) cells showed that a member of Gα protein family Gαq promoted PI3K/AKT signaling pathway through focal adhesion kinase (FAK) for cell growth and survival [130].

Combinatorial CRISPR screening with scRNA-seq showed that driver gene alterations influenced TSGs, and triggered tumorigenesis in human mammary epithelial cells, indicating the impact of transcriptional epistasis on oncogenic mediators and potential therapeutic targets, including CDK4, SRPK1, and DNMT1 [131]. By analyzing the CRISPR-Cas9 screening data from Depmap (Cancer Dependency Map) and TCGA data of differentially expressed genes, the cell cycle pathway was identified as a key pathway of cell viability regulation in breast cancer patients [154]. The CRISPR/Cas9 screening in chemo-resistant small-cell lung cancer (SCLC) identified serine/threonine kinase cell division cycle 7 (CDC7) as a potential synergistic target. Combination of CDC7 inhibitor XL413 and chemotherapy led to apoptosis of chemo-sensitive SCLC in xenograft tumor [132]. Acute Myeloid Leukemia (AML) cell lines such as MOLM-13, MV4-11, HL-60, OCI-AML2, OCI-AML3 were examined for therapeutic targets via genome-wide CRISPR screening, indicating KAT2A inhibition as a therapeutic strategy in AML [133].

Hepatocellular carcinoma (HCC) was examined via CRISPRa for growth and metastasis driver genes. High MYADML2 protein level reduced sensitivity to chemotherapeutic drugs and led to worse survival [134]. Essential single nucleotide polymorphisms (SNPs) for PrCa proliferation were explored via dCas9-KRAB negative screening with 2166 candidate SNP sites in 9133 gRNAs. RIGOR program analysis identified 117 SNPs which tended to reside near 5 kb flanking the transcription start sites. SNP (rs60464856) site targeting in stable dCas9 expressing cell line showed significant down regulation of *RUVBL1* gene, and further validation showed that *RUVBL1* was associated with tumorigenesis [135]. dCas9-KRAB perturbation genome screening identified 470 high-confidence cis enhancer-gene pairs in 5920 enhancers in chronic myelogenous leukemia cell K562, facilitating the large-scale mapping of enhancer-gene regulatory interaction network [49].

#### The utilization of CRISPR-Cas9 in investigating drug resistance against tumors

Resistance to nucleotide analog 6- thioguanine was examined by genome-scale knockout screen in two human cell lines, identified DNA mismatch repair pathway, DNA topoisomerase II (TOP2A) and cyclin-dependent kinase 6, (CDK) for DNA topoisomerase II (TOP2A) poison etoposide, demonstrating Cas9/ sgRNA screens as a powerful tool for systematic genetic analysis in mammalian cells [26]. CRISPR knockout screening in human A549 lung adenocarcinoma cells identified 5 EGF-resistance genes, and further RNAi validation showed DUSP1 increased survival of EGF treated cells, providing a novel target for EGFR-overexpressing cancers [86]. Genome-wide knockout screening using CRISPR-Cas9 was also carried out in respiratory cancers, including Nasopharyngeal carcinoma (NPC) and lung cancer (LC). Nine genes were found to be associated with radiosensitivity of NPC cells (C666-1R, 6-8FR). Fanconi anemia pathway and the TGF-β signaling pathway were reported to be important contributors for radiosensitivity [136]. In the nervous system, neuroblastoma tumorigenesis was investigated via CRISPR genome-wide knockout screening, showed that ubiquitin-specific proteases (USPs) stabilize and increase half-life of repressor element-1 silencing transcription factor (REST), indicating its critical role in neuroblastoma generation [137]. As for reproductive cancers, drug resistance genes as well as lethal genes for cancer cell were identified. Genome-scale screening in ovarian cancer cell lines with the GeCKO library identified one previously validated gene SULF1 and a novel gene ZNF587B responsible for cisplatin resistance [138]. Cervical cancer cell lines such as Hela and Siha were incubated with cisplatin or paclitaxel, respectively, and screened by genome-scale CRISPR/Cas knockout library and ninety-seven genes were identified to be associated with drug resistance [139]. Prostate cancer (PrCa) is one of the most lethal causes of cancer-related death in males. Resistance to Enzalutamide, docetaxel, and Cabazitaxel in metastatic castration-resistant prostate cancer (mCRPC) is a big obstacle for cancer treatment of male patients. Whole-genome CRISPR/Cas9 knockout screening in mCRPC cell line C4 dissected the potential genes responsible for drug resistance. Two genes (IP6K2, XPO4) were validated after the screening process via bioinformatic prediction, highlighting the necessity to perform individualized validation [140].

Phase III clinical trial for Aurora-A (AURKA) inhibitor alisertib (MLN8237) in breast cancer failed to prolong patients’ survival. Rational drug combinations for better therapeutic outcome were carried out based on CRISPR/Cas9 knockout screening of 507 kinases, identifying synthetic lethality interactions with MLN8237 and Haspin (GSG2). The combination of MLN8237 and Haspin inhibitor CHR-6494 reduced tumor growth both in vitro and in vivo [141]. CRISPR screening for 656 E3 ubiquitin ligases in PrCa cells identified 51 genes as tumor repressors. The novel oncodriver Ring Finger Protein 19 A (RNF19A) was frequently amplified and highly expressed in PrCa. It correlated with castration resistance and ubiquitylated Thyroid Hormone Receptor Interactor 13 (TRIP13) and was activated by androgen receptor (AR), and Hypoxia Inducible Factor 1 Subunit Alpha (HIF1A), indicating AR/HIF1A-RNF19A-TRIP13 signaling axis for PrCa therapy [142].

Colorectal cancer (CRC) was examined for drug resistance to oxaliplatin and screened by CRISPR/Cas9 genome-wide library knockdown system. It found that low expression of mitochondrial elongation factor 2 (MIEF2) contributed to oxaliplatin drug resistance by reducing mitochondrial stability and inhibiting apoptosis via decreased cytochrome C release [143]. The CRISPRa system was employed to investigate genes associated with resistance to lymphoma radiotherapy, and a total of 8 genes were screened and subsequently validated, demonstrating a significant correlation with radiotherapy resistance [144]. Patients with Cisplatin-resistant Testicular Germ Cell Tumors (TGCTs) have poor prognosis, and developments of novel therapeutic strategies are critical. CRISPRa system revealed that NEDD8-activating enzyme E1 (NAE1) was highly expressed in drug-resistant colonies of TGCT cells, and indicated that neddylation inhibitor (MLN4924) combined with cisplatin as a novel treatment option for TGCTs [145].

#### Utilizing CRISPR/Cas9 screening for personalized drug selection through patient-derived organoids

Organoids derived from both healthy and diseased tissues offer a valuable resource for biological or pathological investigations. Although CRISPR screening showed powerful manipulation in cancer cells lines, it is also employed for tumor organoids derived from diverse cancer patients for personalized drug selection. Suspension culture increases efficiency of culturing cancer organoids for genome-wide CRISPR-Cas9 screening and large-scale perturbation screens [146]. Human fetal hepatocyte organoids were generated to model nonalcoholic fatty liver disease (NAFLD), and CRISPR screening was utilized to identify steatosis modulators in APOB^−/−^ and MTTP^−/−^ organoids [147]. CRISPR-Cas9 genetic intervention and high-throughput drug screening have been applied in digestive organoids for personalized disease modeling and therapy [155]. Human Pancreatic cancer organoid biobank established from 31 distinct tumor lines was used for CRISPR/Cas9 genome editing and drug screening, indicated increased sensitivity of kinase inhibitors dasatinib and VE-821 with driver gene *ARID1A* mutation [148]. Drug response evaluation by in vivo CRISPR screening (DREBIC) method was used in pancreatic ductal adenocarcinoma organoid [127].

### CRISPR screening in vivo for cancer therapy

In 2022, FDA approved a total of five CAR-T cell products for the treatment of B cell acute lymphoblastic leukemia or high-grade lymphomas, as well as multiple myeloma using lentiviral or γ-retroviral approaches [156]. Notably, two clinical trials (NCT05143307/NCT03872479) employed AAV as the delivery method in their studies on cancer therapy in vivo based on CAR-T cells and CRISPR/Cas system [117]. CRISPR screening system provides a robust genetic tool for in vivo elucidation of CAR-T resistance mechanisms. Loss-of-function genetic screens in an immunocompetent murine model with B-cell acute lymphoblastic leukemia (B-ALL) identified the IFNR/JAK/STAT signaling and antigen processing and presentation pathway as key factors for CAR-T resistance in vivo. In addition, natural killer (NK) cells also engage in the resistance progress [157]. Gain-of-function CRISPR activation screen in primary CD8 + T cells identified a key factor PRODH2 for improving the in vivo efficacy of CAR-T based cell killing. Augmentation of PRODH2 enhanced metabolic function of CAR-T cells as an immune booster [158].

CRISPR screening was also utilized for in vivo investigation to elucidate gene function within a whole organism or the context of complex biological systems, using lentiviral or AAV mediated sgRNA transfection in living organisms. AAV was the widely used vector for in vivo genetic therapy due to its low immunogenicity and non-pathogenic character [118]. The limitation of AAV’s vector capacity has been addressed through the recent development of a two-split intern vectors system [159], while smaller SpCas9 orthologues such as SaCas9 have demonstrated comparable editing efficiency to that of SpCas9, rendering them suitable for AAV-SaCas9 mediated in vivo genome editing [21]. Additionally, Cre-dependent and constitutive Cas9 expressing transgenic mice were established with EGFP labeling, which provides an animal model for genome-wide targeting and contributes to in vivo investigation [24].

In vivo screens were performed in mouse brain, liver, pancreases, lung and so on. The application of SpCas9 and gRNAs using AAV vectors enabled multiple gene modifications in the adult mouse brain, demonstrating its potential for genetic regulation [33]. Gliomagenesis suppressors were investigated by in vivo stereotaxic injection of AAV carrier sgRNA library in conditional-Cas9 mouse brain [160]. Autochthonous invasion of AAV-mTSGs library in Cre-inducible Cas9 mice liver led to cancer development in situ, and the mice died within 4 months [89]. NIT1 cells (a non-obese-diabetic-derived mouse beta cell line) mutated with GeCKO-v2 were subcutaneously transplanted into type 1 diabetes mouse model to identify genes contributing to autoimmune killing resistance [161]. With the AAV9-LPL gene delivery into the lung, multiple mutations of *KRAS*^*G12D*^, *p53* and *LKB1* were obtained to induce macroscopic tumors. In vivo screening for lung cancer TSGs through CRISPR/Cas9 genome-wide knockout showed that ZNF24 contributed to P65 suppression via NF-κB pathway. Combinational inhibition of KRAS, NF-κB, and PD-1 effectively shrank autochthonous Kras^G12D^/ZNF24^−/−^ lung cancers in mouse [162].

Examination of immunotherapy-treated normal and *Tcra*-/- mice in vivo by CRISPR screening showed the loss of CD47 caused resistance to immunotherapy. Deletion of protein tyrosine phosphatase (PTPN2) increased immunotherapy efficacy [88]. CRISPR screening identified PD-1, Tim-3, and RNA helicase Dhx37 as regulators of tumor infiltration and degranulation. Depletion of Dhx37 improved CD8 T cells efficacy towards triple-negative breast cancer in vivo, and the NF-kB signal pathway was involved in the process [163]. In vivo applications of CRISPR screening system were summarized in the following Table 3.

**Table 3:** CRISPR screening in vivo for cancer therapy

Vector type	Cas enzyme	gRNAs library	Target cells	Screening	Application		Reference	
AAV	Cas9	Multiplex genome targeting.	Mouse brain	CRISPRko		Gene function		[33]
AAV9	Cre-inducible Cas9	AAV-mTSG library	Mouse liver	CRISPRko		Tumor suppressors		[88]
Lentiviral	Cas9	Genome-wide sgRNA SKY library	Mouse hemopoietic system	CRISPRko		Intrinsic determinants		[157]
Lentiviral	dCas9	dgRNA library	Primary CD8 + T cells	CRISPRa		genome-scale GOF screen		[158]
AAV1/AAV2 /lentiviral	Cas9	AAV-mTSG library	Mouse brain	CRISPRko		Gene suppressors		[160]
Lentiviral	Cas9	Addgene #1,000,000,052	Type 1 diabetes mouse model	CRISPRko		Protection gene screening		[161]
Lentiviral	Cas9	Addgene #73,178	Lung cancer cell line-EKVX	CRISPRko		TSG genes		[162]
Lentiviral	Cas9	Genome-scale MKO library	CD8 T cells	CRISPRko		Immunotherapy target		[163]

## Limitations and prospection

The advances of CRISPR/Cas technology and screening strategies have revolutionized genetic identification, enabling the dissection of functional genes in specific biological processes and diseases, facilitating drug selection and individualized therapy. CRISPR screening has demonstrated great potential in cancer therapy by offering methods to combat drug resistance and aggressive behaviors, as well as identifying possible gene targets for novel approaches to treat cancers. However, there are still several obstacles for CRISPR/Cas application in clinical cancer treatment, including delivery of CRISPR/Cas9 system, Off-target effect, PAM limitation, as well as multiple gene-editing [117]. In this part, we paid more attention on limitations of CRISPR screening system and CAR-T cell therapy for cancers.

### Limitations of CRISPR screening system

CRISPR screening delivery primarily relies on lentiviral and AAV vectors, which are crucial tools for either ex vivo or in vivo investigation. Of which, AAV vector has the advantages with mildly immunogenic and long-term transgene expression in post-mitotic cells, making it a leading platform for in vivo cancer therapy [164]. However, AAV vector showed some drawbacks in manufacturing, packaging size limitation, vector quality control and editing specificity, as described in the recent review [118].

Except the delivery limitations, the occurrence of off-target effects and unintended mutations induced by CRISPR technology are barriers to its application in clinical therapy. SpCas9 protein showed the ability to identify PAM sequence and cut specific DNA region in the CRISPR system. Due to the tolerance of gRNA recognition and nucleotide indels in the target region, even a single guide can generate thousands of off-targets as detected by sensitive high-throughput sequencing methods such as GUIDE-Seq and CIRCLE-seq [138, 143]. This raises concerns regarding the application of CRISPR technology in gene therapy [165]. The reason of the off-target effect is the conformational states of HNH domain. The activated conformation of HNH increases DNA cleavage efficiency for DNA double-strand break formation, leading to both on- and off-target effects [166]. To minimize the probability of off-target mutagenesis, other high-fidelity nucleases such as SpCas9-HF1, eSpCas9 and HypaCas9 were developed [167, 168]. In addition, PAM sequence limitation for Cas9 has been broadened by the identification of KKH SaCas9 variant, which exhibits robust genome editing activities with the PAM (NNNRRT) while maintaining comparable levels of off-target effects [169].

Anti-CRISPR is another obstacle to overcome because of the restriction of targeting specificity and activities. The VI-CRISPR inhibitors acrVIA1-7 from phage exhibit the ability to block Cas13a RNA targeting and dCas13a-mediated single nucleic acid editing. Specifically, AcrVIA1, 4, 5 and 6 bind to LwaCas13a, while AcrVIA2 and 3 interact with LwaCas13-crRNA complex [170].

### Limitations of CAR-T cancer therapy

Although CAR-T showed success of B-cell malignance treatment, its usage in solid tumors still have some limitations such as T-cell exhaustion, lack of CAR-T cell persistence, and cytokine-related toxicities. To address these challenges, CRISPR technology has been used to generate safe and potent allogeneic universal CAR-T cell products for cancer immunotherapy [152]. However, hurdles remain for solid tumor CAR-T therapy due to target antigen heterogeneity, unable to pass through vascular endothelium to target tumor cells, and the immunosuppressive tumor microenvironments [171]. As viral vectors are commonly used for delivering CAR-T cells, safety concerns have arisen. To address this issue, virus-free CRISPR-CAR (VFC-CAR) T cells were generated [172]. Virus-free CAR-T cells (PD1-19bbz) were generated and a clinical trial was performed and registered at www.clinicaltrials.gov (NCT04213469) [173].

## Future perspectives

Given the capacity of CRISPR to precisely modify the human genome in cells, ethical considerations have emerged as a pivotal factor for its application in genetic manipulation [174–176]. The challenges posed by off-target effects and unintended mutations serve as barriers to the clinical implementation of CRISPR technology. However, extensive efforts have been made to mitigate these concerns through the development of novel strategies, rendering CRISPR technologies indispensable tools for elucidating gene functions and noncoding elements involved in tumorigenesis, as well as facilitating the creation of next-generation cancer immunotherapies. In summary, CRISPR/Cas system continues to play an essential role in advancing human cancer research and clinical therapy.

## Data Availability

Data sharing is not applicable to this article as no datasets were generated or analyzed during the current study.

## List of abbreviations

CRISPR: Clustered regularly interspaced short palindromic repeats
Cas9: CRISPR-associated protein 9
gRNA: Guide RNA
SpCas9: *Streptococcus pyogenes* Cas9
SaCas9: *Staphylococcus aureus* Cas9
StrCas9: *Streptococcus thermophilus* Cas9
Cpf1: CRISPR from *Prevotella* and *Francisella 1*
AAV: Adeno-associated vector
crRNA: CRISPR RNA
tracrRNA: Trans-activating crRNA
PAM: Protospacer adjacent motif
RNP: Ribonucleoprotein
CFTR: Cystic fibrosis transmembrane conductance regulator
nCas9: Cas9 nickase
ABEs: Adenine base-editors
CBEs: Cytosine base-editors
IH-HR: Interhomolog homologous recombination
dCas9: Dead Cas9
RuvC: CDD conserved protein domain family
HNH: Conserved Protein Domain Family HNH, His-Asn-His (HNH)
CRISPRi: CRISPR inhibition
CRISPRa: CRISPR activation
KRAB: Krüppel-associated box
TetO: Tetracycline Inducible Expression promoter
RNAP: RNA polymerase
VP64: Transcriptional activator consists of four copies of VP16
AsCpf1: Cpf1 enzyme from *Acidominococcus sp. BV3L6*
LbCpf1: Cpf1 enzyme from *Lachnospiraceae bacterium ND2006*
HDR: Homology-directed repair
BhCas12b: Cas12b enzyme from *Bacillus hisashii*
SHERLOCK: Specific High-Sensitivity Enzymatic Reporter UnLOCKing
SARS-CoV-2: SARS-coronavirus-2
ORF: Open reading frame
cfDNA: Cell free DNA
PRRSV: porcine reproductive and respiratory syndrome virus
CHIKV: RNA viruses’ chikungunya
dCas13b: Catalytically inactive Cas13b
REPAIR: Programmable A to I Replacement
TKO: Toronto KnockOut
GeCKOv1: Genome-scale CRISPR-Cas9 knockout version 1
MAGeCK: Model-based Analysis of Genome-wide CRISPR/Cas9 Knockout
SAM: Synthetic activation mediator
TSS: Transcription start site
eQTL: Expression quantitative trait locus
TCGA: The Cancer Genome Atlas
MAGeCK: Genome-wide CRISPR/Cas9 Knockout
IDLV: Integration deficient lentiviral
HTGTS: High-throughput genome-wide translocation sequencing
BLESS: Breaks labeling, enrichment on streptavidin and next-generation sequencing
GUIDE-seq: Genome-wide unbiased identification of DSBs enabled by sequencing
DISCOVER-seq: Discovery of in situ Cas off-targets and verification by sequencing
Digenome-seq: Digested genome sequencing
SITE-Seq: Selective enrichment and identification of adapter-tagged DNA ends by sequencing
CIRCLE-seq: Circularization for in vitro reporting of cleavage effects by sequencing
CHANGE-seq: Circularization for high-throughput analysis of nuclease genome-wide ffects by sequencing
CAR: Chimeric antigen receptor
TIL: Tumor-infiltrating lymphocytes
GSCs: Glioblastoma (GBM) stem cells
PD-1: Programmed Cell Death Ligand 1
ESCC: Esophageal squamous cell carcinoma
MEST: Mesoderm Specific Transcript
ZNF: Zinc finger protein
HCC: Hepatocellular carcinoma
PC: Pyruvate carboxylase
EOC: Epithelial ovarian cancer
GNAQ: Guanine nucleotide-binding protein G(q) subunit alpha
UM: Uveal melanoma
FAK: Focal adhesion kinase
TSGs: Tumor suppressor genes
Depmap: Cancer Dependency Map
SCLC: Chemo-resistant small-cell lung cancer
CDC7: Serine/threonine kinase cell division cycle 7
AML: Acute Myeloid Leukemia
HCC: Hepatocellular carcinoma
SNPs: Single nucleotide polymorphisms
TOP2A: DNA topoisomerase II
CDK6: Cyclin-dependent kinase 6
TOP2A: DNA topoisomerase II
NPC: Nasopharyngeal carcinoma
LC: Lung cancer
USPs: Ubiquitin-specific proteases
REST: Repressor element-1 silencing transcription factor
PrCa: Prostate cancer
CRC: Colorectal cancer
MIEF2: Mitochondrial elongation factor 2
NAE1: NEDD8-activating enzyme E1
NAFLD: Nonalcoholic fatty liver disease
B-ALL: B-cell acute lymphoblastic leukemia
PTPN2: Protein tyrosine phosphatase
